# Characterization of the Multi-Drug Resistance Gene *cfr* in Methicillin-Resistant *Staphylococcus aureus* (MRSA) Strains Isolated From Animals and Humans in China

**DOI:** 10.3389/fmicb.2018.02925

**Published:** 2018-11-27

**Authors:** Shu-Min Li, Yu-Feng Zhou, Liang Li, Liang-Xing Fang, Jia-Hong Duan, Fan-Rui Liu, Hua-Qing Liang, Yu-Ting Wu, Wei-Qi Gu, Xiao-Ping Liao, Jian Sun, Yan-Qiong Xiong, Ya-Hong Liu

**Affiliations:** ^1^Laboratory of Veterinary Pharmacology, College of Veterinary Medicine, South China Agricultural University, Guangzhou, China; ^2^LABioMed at Harbor-UCLA Medical Center, Torrance, CA, United States; ^3^Geffen School of Medicine at UCLA, Los Angeles, CA, United States

**Keywords:** *cfr*, MRSA, multi-drug resistance, plasmid, food animals

## Abstract

We investigated *cfr*-positive and -negative MRSA strains isolated from animals and humans in different geographical areas of China, from 2011 to 2016. Twenty *cfr*-positive strains (15.6%) were identified from 128 MRSA strains including 17 from food animals and three from humans. The resistance rates and prevalence of the tested antibiotic resistance genes (ARGs) in the *cfr*-positive MRSA isolates were higher than that in the *cfr*-negative MRSA isolates. All *cfr*-positive MRSA isolates were co-carrying *fexA* and *ermC*, and had significantly higher *optrA* incidence rate vs. the *cfr*-negative isolates (*P* < 0.05). In addition, multilocus sequence typing (MLST) assays showed that ST9 and *spa*-type t899 were the most prevalent ST and *spa* types in the study strains. However, all of the 20 *cfr*-positive and 10 randomly selected *cfr*-negative MRSA isolates were clonally unrelated as determined by pulsed-field gel electrophoresis (PFGE) analyses. Importantly, the *cfr* gene was successfully transferred to a recipient *Staphylococcus aureus* strain RN4220 from 13 of the 20 *cfr*-positive MRSA isolates by electroporation. Among these 13 *cfr*-positive MRSA isolates, two different genetic contexts surrounding *cfr* were determined and each was associated with one type of *cfr*-carrying plasmids. Of note, the predominant genetic context of *cfr* was found to be a Tn*558* variant and locate on large plasmids (∼50 kb) co-harboring *fexA* in 11 of the 13 MRSA isolates. Furthermore, the *cfr* gene was also identified on small plasmids (∼ 7.1 kb) that co-carried *ermC* in two of the 13 MRSA isolates. Our results demonstrated a high occurrence of multi-drug resistance in *cfr*-positive MRSA isolates, and the spread of *cfr* might be attributed to horizontal dissemination of similar *cfr*-carrying transposons and plasmids.

## Introduction

The chloramphenicol–florfenicol resistance (*cfr*) gene encodes a methyltransferase that modifies position A-2503 in bacterial 23S rRNA and confers resistance to five classes of antibiotics (phenicols, lincosamides, oxazolidinones, pleuromutilins, and streptogramin A) (Long et al., 2006; Morales et al., 2010). These antibiotics have been widely used for the treatment of infections in human and animal (Inkster et al., 2017; Li J. et al., 2017). Since the first identification of the *cfr* gene in *Staphylococcus sciuri* isolates in 2000, it has been subsequently found in *Enterococcus* spp., *Bacillus* spp., *Streptococcus suis*, *Proteus vulgaris*, and *Escherichia coli* (Long et al., 2006; Wang et al., 2011, 2012a,b). In China, most *cfr*-positive isolates were derived from domestic animals (mainly pigs). In addition, plasmids and insertion sequences were implicated in *cfr* gene dissemination between species and genera (Shen et al., 2013).

Methicillin-resistant *Staphylococcus aureus* (MRSA) can cause a wide range of infections, including skin and soft-tissue infections as well as endocarditis and respiratory tract infections (Marshall and McBryde, 2014; Rodvold and McConeghy, 2014). Hospital-acquired MRSA (HA-MRSA) and community-acquired MRSA (CA-MRSA) are the primary origins for infections in humans (Woodford and Livermore, 2009). However, livestock-acquired MRSA (LA-MRSA) have been identified in pigs, ducks, poultry, and rats (Voss et al., 2005; Wulf et al., 2006; de Neeling et al., 2007; van de Giessen et al., 2009). Importantly, LA-MRSA containing the plasmid-borne *cfr* gene has been identified in infections of farmers suggesting zoonotic transmission (Wulf et al., 2006; Cui et al., 2009).

In this study, we investigated the epidemiological characteristics and dissemination of the *cfr* gene in clinical MRSA isolates from animal and human sources. We compared the phenotypic and genotypic profiles of *cfr*-positive MRSA strains with *cfr*-negative MRSA strains.

## Materials and Methods

In total, 128 MRSA strains were isolated from pigs, chickens, and ducks in 10 different regions of China and from clinical patients in two different hospitals in Guangzhou, China during 2011–2016. All MRSA isolates were confirmed by MALDI-TOF/MS system (Shimadzu-Biotech, Japan), multiplex PCR amplification, and DNA sequencing of the *mecA* gene.

Minimum inhibitory concentrations (MIC) were determined using a standard agar dilution method -CLSI M100-S28 and VET01-A4/VET01-S2. The tested antibiotics were phenicols (florfenicol), lincosamides (clindamycin), oxazolidinones (linezolid), pleuromutilins (valnemulin), β-lactams (ampicillin and cefotaxime), macrolides (tylosin, azithromycin, and erythromycin) and ciprofloxacin, gentamycin, tetracycline, rifampicin, trimethoprim-sulfamethoxazole, vancomycin, and daptomycin. The MIC breakpoints of each antibiotic against MRSA were used as recommended by the current CLSI guidance (Clinical and Laboratory Standards Institute [CLSI], 2013, 2018). *S. aureus* ATCC 29213 was used as a quality control strain.

### Detection of Resistance Genes

The presence of the *cfr* gene in the MRSA strains was determined with PCR as described previously (Kehrenberg and Schwarz, 2006). Other genes that encoded resistance to phenicols (*fexA*), lincomycin [*lnu*(A), (F)], oxazolidinones (*optrA*), pleuromutilins (*vgaAV)*, macrolide–lincosamide–streptogramin B (*ermA-C*), macrolides (*ereA-B*), tetracycline [*tet*(A), (C), (L), (M), and (K)]. and aminoglycosides [*aac(3*′*)-Ia, aac(3*′*)-IIc*, *aadA1*, *aadB*, *aph(3*′*)-II*, *aph(3*′*)-IV*, *aph(4*′*)-Ia*, and *aac(6*′*)-Ib*] were identified by PCR using gene-specific primers (Supplementary Table S1).

### Molecular Typing

Genetic diversity of *cfr*-positive and -negative MRSA isolates was determined by *SmaI* pulsed-field gel electrophoresis (PFGE) (Tenover et al., 1995). Comparison of PFGE patterns was performed with BioNumerics software (Applied Maths, Sint-Martens-Latem, Belgium). Dendrograms were generated using Dice similarity coefficient and analogical values to categorize identical PFGE types cut-offs were fixed at 100%. Further determinations of clonality were performed by multilocus sequence typing (MLST) and *spa* typing as described previously.^1^^,^^2^ *Salmonella enterica* serotype Braenderup H9812 DNA was used as a molecular size marker (Tenover et al., 1995).

### Transformation of *cfr* Gene and Determination of *cfr* Location

Plasmid DNA from *cfr*-positive MRSA strains was extracted using a Qiagen Prep Plasmid Midi Kit (Qiagen, Hilden, Germany) and transferred into a recipient *S. aureus* strain RN4220 by electroporation using Gene Pulser apparatus (Bio-Rad, Hercules, CA, United States). Electrotransformants were selected on brain heart infusion (BHI) agar containing 8 μg/mL of florfenicol. The presence of *cfr* was further confirmed by PCR (Kehrenberg et al., 2009). To determine the location of *cfr* gene, DNA was separated by PFGE after treatment with *S1* nuclease (Takara, Dalian, China) and plasmids carrying *cfr* were identified by Southern blot hybridization using a digoxigenin-labeled *cfr* probe (Roche, Mannheim, Germany) according to the manufacturer’s instruction.

### Genetic Environment of *cfr* Gene

The genetic environment surrounding *cfr* was determined by PCR mapping, inverse PCR, and sequencing (Wang et al., 2015). The primers used to determine the regions upstream and downstream of *cfr* gene and reference sequences containing the *cfr* gene used for PCR mapping are listed in Supplementary Table S2. The obtained DNA sequences were analyzed using BLAST,^3^ and then compared to those deposited in GenBank.

### Statistical Analyses

Statistical significance for the comparison of prevalence data and proportions was determined using a χ^2^ test. *P* < 0.05 was consideredto be statistical significant.

## Results

### Antimicrobial Susceptibility and Presence of Resistance Genes

**FIGURE 1:**
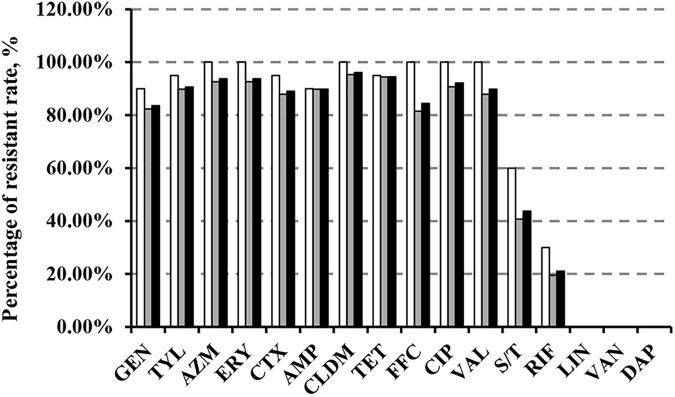
Antibiotic resistance in MRSA strains from animals and humans. GEN, gentamicin; TYL, tylosin; AZM, azithromycin; ERY, erythromycin; CTX, cefotaxime; AMP, ampicillin; CLDM, clindamycin; TET, tetracycline; FFC, florfenicol; CIP, ciprofloxacin; VAL, valnemulin; S/T, sulfamethoxazole/trimethoprim; RIF, rifampicin; LIN, linezolid; VAN, vancomycin; DAP, daptomycin.

We demonstrated that >80% of the 128 MRSA isolates were resistant to all tested antimicrobial agents with the exception of sulfamethozaxole/trimethoprim (43.8%), rifampicin (21.1%), linezolid (1.56%), vancomycin (0%), and daptomycin (0%) (Figure 1). Importantly, resistance rates in *cfr*-positive strains were higher than in *cfr*-negative strains for sulfamethozaxole/trimethoprim (60 vs. 40.7%) and rifampicin (30 vs. 19.4%) (Figure 1). In addition, the proportion of isolates with increased linezolid MIC (≥2 μg/mL) was significantly higher in the *cfr*-positive MRSA vs. the *cfr*-negative MRSA (40 vs. 6.5%, *P* < 0.001) (Supplementary Table S3).

**FIGURE 2:**
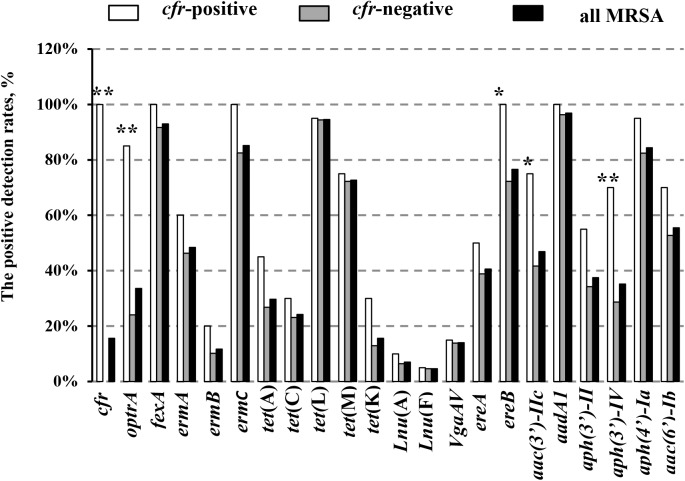
Positive detection rates of genes related to antibiotic resistance in the study MRSA strains. Detection rates between the *cfr*-positive and *cfr*-negative MRSA strains were determined using the χ^2^ test. ^∗^*P* < 0.05; ^∗∗^*P* < 0.01 *cfr*-positive MRSA strains vs. *cfr*-negative MRSA strains.

In addition, 20 of the 128 MRSA strains (15.6%) harbored the *cfr* gene, and included 13 isolates from pigs (10.2%), three from chickens (2.3%), one from duck (0.8%), and three from humans (2.3%). Interestingly, all of the *cfr*-positive MRSA strains also carried the *fexA*, *ermC*, *ereA*, and *aadA1* genes (Table 1). In addition, the prevalence of all the other tested antibiotic resistance genes (ARGs) was higher in the *cfr*-positive MRSA isolates than in *cfr*-negative MRSA isolates, especially for the *optrA*, *ereB*, *aac (3*′*)-IIc*, and *aph (3*′*)-IV* genes (*P* < 0.05; Figure 2).

**Table 1:** Background information and characteristics of *cfr*-positive MRSA.

Strains^a^	ST-*spa*	Year	Source	Resistance profile^b^	Other resistance genes^c^	*cfr* location (size, kb)	*cfr* genetic environmental types
5ZX13	ST9-t7880	2012	Pig	FFC, AMZ, ERY, CLDM, CIP, AMP, CTX, TET, GEN, TYL, RIF, VAL	*fexA, ermC* *optrA*, *ermA*	Plasmid (∼50)	I
5ZB12	ST9-t899	2011	Pig	FFC, AMZ, ERY, CLDM, CIP, TYL, AMP, CTX, TET, GEN, S/T, VAL	*fexA, ermC,* *optrA*	Plasmid (∼50)	I
6ZB3	ST9-t899	2012	Pig	FFC, AMZ, ERY, CLDM, CIP, AMP, CTX, TET, GEN, TYL, S/T, VAL	*fexA*, *ermC*, *optrA*	Plasmid (∼50)	I
2ZG3	ST9-t899	2012	Pig	FFC, AMZ, ERY, CLDM, CIP, TYL, AMP, CTX, TET, GEN, TIG, S/T, VAL	*fexA, ermC*, *ermA*	Plasmid (∼50)	I
2ZX3	ST9-t899	2012	Pig	FFC, AMZ, ERY, CLDM, CIP, AMP, CTX, TET, GEN, TYL, RIF, VAL	*fexA*, *ermC*, *optrA*, *ermA*	Plasmid (∼50)	I
5ZB14	ST9-t899	2012	Pig	FFC, AMZ, ERY, CLDM, TYL, AMP, CTX, TET, GEN, CIP, S/T, VAL	*fexA*, *ermC*, *optrA*	Plasmid (∼50)	I
N3	ST9-t899	2012	Pig	FFC, AMZ, ERY, CLDM, CIP, AMP, CTX, TET, TIG, GEN, TYL, VAL	*fexA*, *ermC*, *opt*r*A*, *ermA*	Plasmid (∼50)	I
25FS35	ST9-t899	2016	Pig	FFC, AMZ, ERY, CLDM, CIP, AMP, CTX, TET, RIF, TYL, S/T, VAL	*fexA*, *ermC*, *optrA*	Plasmid (∼50)	I
HYB6	ST9-t899	2016	Pig	FFC, AMZ, ERY, CLDM, TYL, AMP, TIG,CTX, TET, GEN, CIP, VAL	*fexA*, *ermC*, *optrA*, *ermA*	Plasmid (∼50)	I
YFC28	ST9-t899	2014	Chicken	FFC, AMZ, ERY, CLDM, TYL, TET, GEN, AMP, CTX, RIF, CIP, VAL	*fexA*, *ermC*, *optrA*, *ermA*	Plasmid (∼50)	I
HB119	ST9-t899	2016	Chicken	FFC, AMZ, ERY, CLDM, AMP, CTX, TET, GEN, CIP, TYL, VAL	*fexA*, *ermC*, *ermA*	Plasmid (∼50)	I
25FS24	ST9-t899	2016	Pig	FFC, AMZ, ERY, CLDM, TYL, CTX, TET, RIF, CIP, S/T, VAL	*fexA*, *ermC*, *optrA*	Plasmid (∼7.1)	II
26FS31	ST9-t899	2016	Chicken	FFC, AMZ, ERY, CLDM, TYL, CTX, TET, GEN, CIP, BAC, VAL	*fexA*, *ermC*, *optrA*	Plasmid (∼7.1)	II
6Y2C	ST398-t7829	2012	Duck	AMP, CTX, TET, FFC, GEN, CIP,TYL, AMZ, ERY, RIF, CLDM, S/T, VAL	*fexA*, *ermC, optrA*, *ermA*	ND	
6ZB5	ST9-t899	2012	Pig	AMP, CTX, TET, FFC, GEN, CIP, TYL, TIG, AMZ, ERY, CLDM, VAL	*fexA*, *ermC*, *optrA*, *ermA*	ND	
7SX2	ST9-t899	2012	Pig	AMP, CTX, TET, FFC, GEN, CIP, TYL, AMZ, ERY, CLDM, VAL	*fexA,* *ermC*, *optrA*, *ermA*	ND	
N4-2	ST9-t899	2012	Pig	AMP, CTX, TET, FFC, GEN, CIP, TYL, RIF, AMZ, ERY, CLDM, S/T, VAL	*fexA*, *ermC*, *optrA*, *ermA*	ND	
BA13	ST9-t899	2016	Human	AMP, CTX, TET, FFC, GEN, CIP, TYL, RIF, AMZ, ERY, CLDM, S/T, VAL	*fexA*, *ermC*, *optrA*	ND	
161429	ST764-t1084	2016	Human	AMP, CTX, TET, FFC, GEN, CIP, TYL, AMZ, ERY, CLDM, S/T, VAL	*fexA*, *ermC*, *optrA*, *ermA*	ND	
161494	ST764-t1084	2016	Human	AMP, CTX, TET, FFC, GEN, CIP, TYL, AMZ, ERY, CLDM, VAL	*fexA*, *ermC*, *optrA*, *ermA*	ND	

*^a^Isolates from electrotransformants are underlined. ^b^Resistance profiles of transferred strains are underlined. AMP, ampicillin; CTX, cefotaxime; TET, tetracycline; FFC, florfenicol; GEN, gentamicin; CIP, ciprofloxacin; TYL, tylosin; AZM, azithromycin; ERY, erythromycin; CLDM, clindamycin; S/T, sulfamethoxazole/trimethoprim; RIF, rifampicin; VAL, valnemulin. ^c^Genes that were co-transferred with cfr are underlined. ND, not determined.*

### Molecular Typing

The 128 MRSA strains contained eight ST types and seven *spa* types, and ST9 (82.0%, 105/128) and *spa* type t899 (80.5%, 103/128) were predominated. In the *cfr*-positive MRSA isolates, three ST types and four *spa* types were observed, and ST9 (85%, 17/20) and *spa* type t899 (75%, 15/20) were also the most prevalent of these types. We observed 12 different profiles using a combination of MLST and *spa* typing in the 128 MRSA isolates. ST9-t899 (78.9%, 101/128) and ST764-t1084 (6.3%, 8/128) were the most and second most ST-*spa* types, respectively (Supplementary Table S4).

**FIGURE 3:**
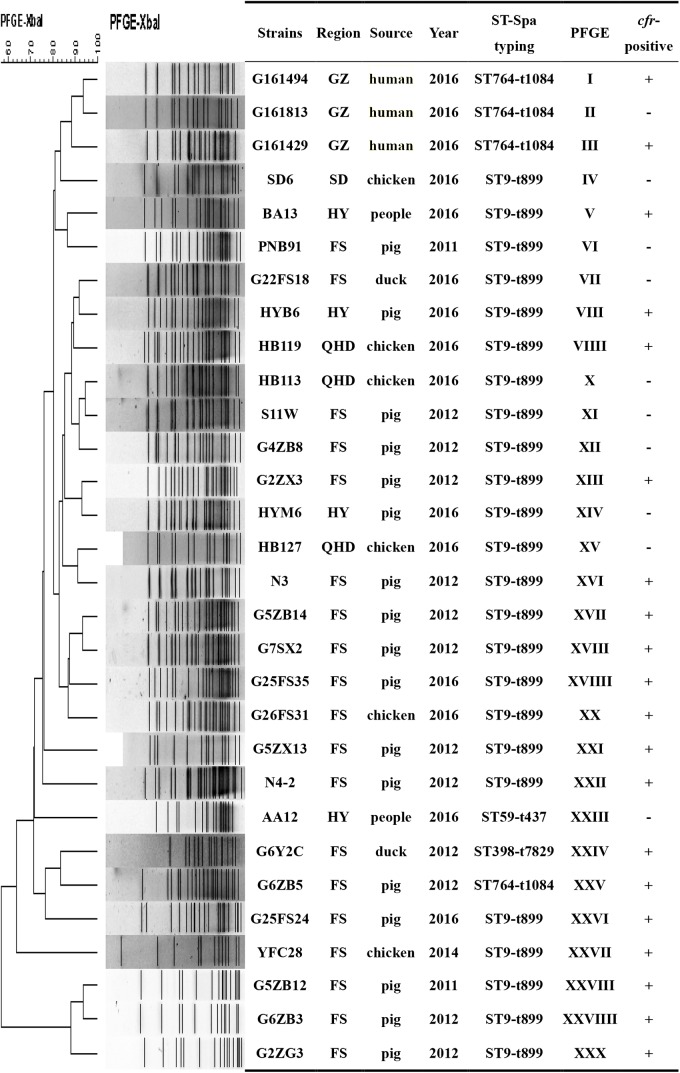
PFGE fingerprint patterns of *SmaI*-digested total DNA preparations from 20 MRSA strains harboring *cfr* and 10 *cfr*-negatives MRSA strains. A similarity cutoff of 100% was used to identify a PFGE cluster. Guangzhou (GZ), Qinhuangdao (QHD), Foshan (FS), Shandong (SD), Huadu (HD), and Heyuan (HY). “+”, *cfr*-positive; “–” <LIST>*cfr*-negative.

We also found 20 different PFGE profiles in the *cfr*-positive and 10 *cfr*-negative MRSA isolates (Figure 3). PFGE analysis suggested that MRSA isolates in the current study were epidemiologically unrelated clones.

### Transfer of *cfr* and Plasmids Analyses

The *cfr* gene from 13 of the 20 *cfr*-positive MRSA isolates were successfully transferred to a recipient strain (*S. aureus* RN4220) and showed 4- to 64-fold increases in the MICs of florfenicol as compared with the recipient strain lacking the *cfr* gene. In addition, *cfr* gene transfenerated strains were resistant to erythromycin, azithromycin, and clindamycin. Co-transfer of *cfr* with *fexA* and *ermC* genes were found in eight of 13 electrotransformants. *S1*-PFGE and Southern blot hybridizations revealed that the *cfr* genes were located on plasmids with sizes of 50 kb (*n* = 11) or 7.1 kb (*n* = 2) (Table 1).

### Genetic Environment of *cfr* Gene

**FIGURE 4:**
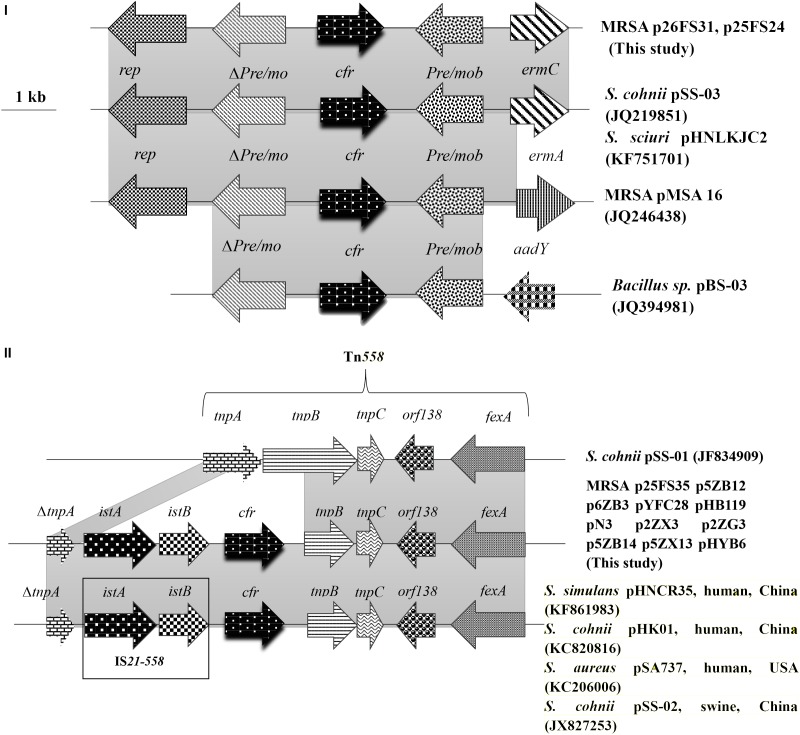
The genetic context surrounding the *cfr* gene in plasmids and their structural comparison with plasmids possessing have >98% similarity. The arrows indicate the positions and directions of the transcription of each gene. Gray shaded regions indicate homology >98%. “Delta” represents a truncated gene.

The genomic structure surrounding *cfr* in the 13 *cfr*-carrying electrotransformants showed two different genetic contexts. Type I was the most common structure observed in 11 of 13 among which the *cfr* gene was located on ∼ 50 kb plasmids. The 9,880 bp *cfr*-containing regions comprised a truncated *tnpA* (*DeltatnpA*), *istA*, *istB*, *cfr*, *tnpB*, *tnpC*, *orf138*, and *fexA*. This was a Tn*558* variant with a 5′ deletion of *tnpB* by insertion of the IS*21-558* element (*istA-B*) and *cfr* in the same orientation. Type II was similar to that in plasmid pHNCR35 (KF861983), pSS-02 (JX827253), pHK01 (KC820816), and pSA737 (KC206006). Type II was found in two electrotransformants among which the complete nucleotide sequences of 7,057-bp circular plasmids harboring *cfr* (p26FS31 and p25FS24) were obtained. A plasmid comparison based on a BLAST query revealed that p26FS31 and p25FS24 were identical to plasmid pSS-03 (JQ219851) and pHNLKJC2 (KF751701). These plasmids consisted of five open reading frames (ORF) (*rep*-Delta*pre/mob-cfr-pre/mob*-*ermC*) (Figure 4).

## Discussion

In this study, we investigated the prevalence of *cfr* in 128 MRSA strains isolated from animals and humans in China. Our study showed a significantly higher positive rate of *cfr* in LA-MRSA strains from animals (15.17%) than that recently reported in domestic studies (1.11–3.46%) (Li et al., 2015; Li J. et al., 2017). In addition, *cfr* was also present in one MRSA isolate from domestic duck. To the best of our knowledge, this is the first report on the *cfr* gene in MRSA strains from waterfowl. This finding may implicate a recent and rapid dissemination process of *cfr* in MRSA strains from different food animals in China. Moreover, the prevalence of *cfr* in MRSA strains from humans (2.34%) was higher in the current study than that previously reported for clinical patients (0.30%) (Cai et al., 2015), but lower in isolates from a teaching hospital in a different region of China (9.38%) (Tian et al., 2014).

Most of the *cfr*-positive MRSA strains in the current study presented a multidrug-resistant phenotype and harbored diverse ARGs. These observations were similar to the high occurrence of multidrug resistance previously reported in *cfr*-positive MRSA isolates from swine farms and retail meat in China (Zeng et al., 2014; Li J. et al., 2017). In addition, the *cfr* gene has been reported to be associated with oxazolidinone resistance in several studies (Schwarz et al., 2000; Shen et al., 2013), but it only mediated low levels of resistance to this antibiotic class. In the current study, we found that the proportion of MRSA isolates with increased linezolid MICs in the *cfr*-positive MRSA strains was higher than in the *cfr*-negative MRSA strains. Interestingly, we also determined that the majority of *cfr*-positive MRSA isolates harbored *optrA*, which is in agreement with previous reports suggesting that *optrA* and *cfr* coexist (Li et al., 2016; Fan et al., 2017). In addition to *optrA*, our study *cfr*-positive MRSA isolates also co-carried *fexA* and *ermC*, which is also consistent with previous studies (Liu .X et al., 2017). This linked the cotransmission of *fexA* and *ermA-C* with *cfr* gene in diverse plasmids from coagulase-negative *Staphylococci* as well as *Enterobacteriaceae* of different origins (Wang et al., 2012a, 2013; Ye et al., 2015). Moreover, we observed different ratios of *ermA*, *ermB*, and *ermC* in our study strains that may be related to the location of the genes. For instance, *ermA* and *ermB* are primary chromosomal genes, while *ermC* gene is often plasmid-borne (Schwarz et al., 2011; Kadlec et al., 2012). The *ermB* was present in a minority of our strains, while *ermA* and *ermC* were frequent in MRSA strains (Lina et al., 1999; Liu H. et al., 2017). Furthermore, we found that the majority of the *cfr* genes were located on plasmids. Therefore, these factors may have influenced on the high ratio of *ermC* as we observed in the current studies.

Among all the study MRSA isolates, ST9 and t899 were the most prevalent ST and *spa* types, respectively. ST9 was reported as the predominant ST type in *S. aureus* isolates from animals in China (Cui et al., 2009), and sporadically occurred in Canada, England, Germany, and the United States (Mulders et al., 2010; Fessler et al., 2011; Dhup et al., 2015). In other and our current study, ST9 in *S. aureus* isolates were also found in farmers (Fessler et al., 2011; Dhup et al., 2015; Sun et al., 2015). Emergence of the *cfr* gene in the prevalent ST9 MRSA isolates from pigs and pig-handlers would probably extend the potential reservoirs and expand the risk to human health (Ye et al., 2015; Yan et al., 2016). Since the ST398 was first identified in pigs and pig farmers in 2005 (Armand-Lefevre et al., 2005), it has become the most prevalent MLST-type in LA-MRSA in the United States and Europe (Armand-Lefevre et al., 2005; Cuny et al., 2010; Antoci et al., 2013). More importantly, the ST398 LA-MRSA carrying the *cfr* gene has been detected in Korea and other countries (Kadlec et al., 2012; Moon et al., 2015). Despite the wide and rapid dissemination of *cfr* gene in S. *aureus* isolates in China, to date, *cfr* was only identified in ST398 MRSA isolates from pigs (Li W. et al., 2017).

In the current study, we also found a *cfr*-positive ST398 MRSA strain isolated from a duck indicating a possibility of widespread dissemination of the *cfr*-harboring ST398 LA-MRSA clone in China. In addition, all of the three *cfr*-positive MRSA isolates from patients were identified as ST764, the increased prevalent hybrid variant of the ST5 HA-MRSA lineage with the arginine catabolic mobile element (ACME) in China, Japan, and other Asian areas (Otsuka et al., 2012; Nakaminami et al., 2014; Wang et al., 2016). These results indicated that *cfr*-positive MRSA isolates from animals and humans belonged to different ST types and were probably from epidemiologically unrelated MRSA clones.

In the MRSA isolates from food animals, the *cfr* genes were primarily located on two types of transferable plasmids with sizes of ∼ 50 and ∼ 7.1 kb. Two different genetic contexts surrounding *cfr* were found, and each was associated with one type of *cfr*-carrying plasmid. The predominant genetic context of *cfr* was found to be a Tn*558* variant in the large plasmids that co-carried *fexA*. This suggested that the acquisition of *cfr* could be involved in IS*21-558* mediated recombination. Importantly, the Tn*558* variant also occurred in *Bacillus*, *S. sciuri, Staphylococcus simulans*, and MRSA isolates from humans and swine (Wang et al., 2015; Li J. et al., 2017).

In addition, we also found the *cfr* gene on small plasmids that co-carried *ermC* in MRSA isolates from food animals.These small plasmids were also identified in *Staphylococcus* and *Bacillus* species isolates from pigs (Wang et al., 2012c). The high similarity of the genetic environment of *cfr* among diverse MRSA strains and sources indicated that horizontal transmission mediated by plasmids and transposons played a significant role in dissemination of *cfr*.

## Conclusion

Our studies demonstrated higher antibiotic resistance rates in the *cfr*-positive vs. -negative MRSA isolates. Horizontal transmission mediated by plasmids and transposons likely played an important role in co-dissemination of *cfr* with *fexA* and *ermC*. The transmission of similar *cfr*-carrying transposons and plasmids from diverse bacteria species and origins requires continued investigation.

## Ethics Statement

All procedure of strain isolation from animals was approved by the South China Agriculture University (SCAU) Animal Ethics Committee and conducted in strict accordance with technical guidelines for isolation and identification of animal-origin *Staphylococcus aureus* (DB51/T 2363-2017), as issued by the Quality and Technical Supervision Bureau of China, and in accordance with the SCAU Institutional Animal Care and Use Committee guidelines. The owner of farms from which animal-related samples were taken gave permission for their animals to be used in this study. All strains with human-origin were kindly provided by the Third Affiliated Hospital of Sun Yat-sen University and Guangdong Second Traditional Chinese Medicine Hospital, and the isolation procedure was in accordance with their Institutional Strain Isolation guidelines.

## Author Contributions

Y-HL and Y-QX designed and organized the study. S-ML did the research. J-HD, F-RL, H-QL, Y-TW, and W-QG did the assisted help. LL, L-XF, X-PL, and JS analyzed the data. S-ML and Y-FZ wrote the paper.

## Conflict of Interest Statement

The authors declare that the research was conducted in the absence of any commercial or financial relationships that could be construed as a potential conflict of interest.

## Footnotes

1 http://saureus.mlst.net/

2 http://www.spaserver.ridom.de/

3 https://blast.ncbi.nlm.nih.gov/Blast.cgi
