# Supplementary material for: Characterization of the Multi-Drug Resistance Gene cfr in Methicillin-Resistant Staphylococcus aureus (MRSA) Strains Isolated From Animals and Humans in China
Source: Front Microbiol. 2018 Nov 27;9:2925. doi: 10.3389/fmicb.2018.02925 (PMC6277690; doi:10.3389/fmicb.2018.02925)
Supplement: Supplementary file 1 [file Data_Sheet_1.docx]

**Supplementary data**

**Table S1. Resistance genes related to relevant antibiotics**

| **Antibiotic Classification** | **Representative antibiotics** | **Specific genes** | **References** |
| --- | --- | --- | --- |
| Phenicol | florfenicol | *cfr*, *fexA*, *optrA* | ([Murakami et al., 1991](#_ENREF_6);[Kehrenberg and Schwarz, 2006](#_ENREF_2);[Wang et al., 2015b](#_ENREF_11)) |
| Tetracycline | tetracycline | *tet*(A), *tet*(C), *tet*(L), *tet*(M), *tet*(K), | ([Ng et al., 2001](#_ENREF_7);[Malhotra-Kumar et al., 2005](#_ENREF_5);[Fard et al., 2011](#_ENREF_1)) |
| Lincomycin | clindamycin | *Lnu*(A), *lnu*(F) | ([Lozano et al., 2012](#_ENREF_4)) |
| Macrolide | tylosin, azithromycin, erythromycin | *ereA*, *ereB* | ([Sutcliffe et al., 1996](#_ENREF_8)) |
| MLSB | clindamycin, tylosin, azithromycin, erythromycin | *ermA*, *ermB*, *ermC* | ([Lina et al., 1999](#_ENREF_3)) |
| Aminoglycoside | kanamycin, gentamycin, tobramycin | *aac(3')-IIc*, *aadAI*, *aph(3')-II*, *aph(3')-IV*, *aph(4')-Ia*, *aac(6')-Ib* | ([Vakulenko et al., 2003](#_ENREF_9)) |
| Pleuromulin | valnemulin | *vgaAV* | ([Lina et al., 1999](#_ENREF_3)) |

**Table S2. Primers used for the study of *cfr* surrounded genetic environment**

| **Region** | **Primer name** | **Sequence (5’ to 3’)** | **PCR product length (kb)** | **Reference** | **Reference sequences**  **Acc. No.** |
| --- | --- | --- | --- | --- | --- |
| *cfr-tnpB* | *cfr*-F | TTTATTTGCATTAAGTCCTCGT | 1.62 | This study | KF861983 |
|  | *tnpB*-R | GAATGCTACCGTTATTCGT |  |  |  |
| *tnpB-fexA* | *tnpB*-F | ATGAAGAAAAGATAACGGACA | 1.85 | This study | KF861983 |
|  | *fexA*-R | TACTCATTGCCTTAATAGCTG |  |  |  |
| *istA*-*cfr* | *istA*-F | CGCTGCTTTATGAATCCGAGA | 3.09 | This study | KF861983 |
|  | *cfr*-R | GAATCATTTACACCAGGCAAC |  |  |  |
| *cfr-fexA* | *cfr*-F | TTATATCATGTTGCCTGGTGT | 3.46 | This study | KF861983 |
|  | *fexA*-R | TCAATACGGTCATAATTGCAT |  |  |  |
| *tnpA-cfr* | *tnpA*-F | TTTAAGGGGTTTCTTCACCA | 3.7 | This study | KF861983 |
|  | *cfr*-R | CTTCCCTGATTTATAGCGACT |  |  |  |
| Inverse PCR  for *cfr* | *cfrI*-*F* | ATAGTGAGGAACGCAGCAAAT | 6.31 | ([Wang et al., 2015a](#_ENREF_10)) | JQ219851 |
|  | *cfrI-R* | TCCAATGTCGCCTGTAGCA |  |  |  |

F, forward primer; R, reverse primer.

**Table S3. MIC distribution of linezolid against the study MRSA strains.**

| **Groups (No. of strains)** | **MICs of linezolid（μg/mL）** | | | | | |
| --- | --- | --- | --- | --- | --- | --- |
|  | **<0.5** | **1** | **2** | **4** | **8** | **64** |
| *cfr*-positive MRSA strains (20) | 2(10%) | 12(60%) | 3(15%) | 5(25%) | 0(0%) | 0(0%) |
| *cfr*-negative MRSA strains (108) | 12(11%) | 89(82%) | 6(6%) | 1(1%) | 0(0%) | 0(0%) |
| All study MRSA strains (128) | 14(11%) | 103(80%) | 7(6%) | 4(3%) | 0(0%) | 0(0%) |
| Quality Control Ranges for linezolid | 1~4 | | | | | |
| *Staphylococcus aureus*  ATCC 29213 | 1 | | | | | |

**Table S4. Distribution of ST and *spa* typing in the study MRSA strains.**

| **ST type** | **128 MRSA strain (No. of strains with certain ST/*spa* types)** | **20 *cfr*-positives MRSA strain (No. of strains with certain ST/*spa* types)** | **108 *cfr*-negatives MRSA strains (No. of strains with certain ST/*spa* types))** |
| --- | --- | --- | --- |
| ST9 | 105 (82%) | 17 (85%) | 88 (82%) |
| ST398 | 2 (2%) | 1 (5%) | 1 (1%) |
| ST764 | 9 (7%) | 3 (15%) | 6 (6%) |
| ST59 | 5 (5%) | 0 (0%) | 5 (5%) |
| ST45 | 1 (1%) | 0 (0%) | 1 (1%) |
| ST1376 | 1 (1%) | 0 (0%) | 1 (1%) |
| ST294 | 1 (1%) | 0 (0%) | 1 (1%) |
| ST2483 | 2 (1%) | 0 (0%) | 2 (3%) |
| ***spa* type** |  |  |  |
| t899 | 103 (80%) | 15 (75%) | 88 (81%) |
| t1084 | 9 (7%) | 3 (15%) | 6 (5%) |
| t7829 | 3 (2%) | 1 (5%) | 2 (2%) |
| t437 | 4 (3%) | 0 (0%) | 4 (4%) |
| t1939 | 2 (2%) | 0 (0%) | 2 (2%) |
| t7880 | 2 (2%) | 1 (5%) | 1 (1%) |
| t10119 | 1 (1%) | 0 (0%) | 1 (1%) |
| N.D | 4 (3%) | 0 (0%) | 4 (4%) |
| **ST-*spa*** |  |  |  |
| ST9-t899 | 101 (79%) | 15 (75%) | 86 (80%) |
| ST9-t1939 | 2 (2%) | 0 (0%) | 2 (2%) |
| ST398-t7829 | 1 (1%) | 1 (5%) | 0 (0%) |
| ST764-t1084 | 9 (7%) | 3 (15%) | 6 (6%) |
| ST59-t899 | 1 (1%) | 0 (0%) | 1 (1%) |
| ST1376-t899 | 1 (1%) | 0 (0%) | 1 (1%) |
| ST9-t10119 | 1 (1%) | 0 (0%) | 1 (1%) |
| ST2483-t899 | 2 (2%) | 0 (0%) | 2 (2%) |
| ST398-t7880 | 1 (1%) | 1 (5%) | 0 (0%) |
| ST9-7829 | 1 (1%) | 0 (0%) | 1 (1%) |
| ST63-t899 | 1 (1%) | 0 (0%) | 1 (1%) |
| ST59-t437 | 5(3%) | 0 (0%) | 5(5%) |

**N.D: not determined.**

**Reference**

Fard, R.M., Heuzenroeder, M.W., and Barton, M.D. (2011). Antimicrobial and heavy metal resistance in commensal *enterococci* isolated from pigs. *Vet Microbiol* 148**,** 276-282.

Kehrenberg, C., and Schwarz, S. (2006). Distribution of florfenicol resistance genes *fexA* and *cfr* among chloramphenicol-resistant *Staphylococcus* isolates. *Antimicrob Agents Chemother* 50**,** 1156-1163.

Lina, G., Quaglia, A., Reverdy, M.E., Leclercq, R., Vandenesch, F., and Etienne, J. (1999). Distribution of genes encoding resistance to macrolides, lincosamides, and *Streptogramins* among *Staphylococci*. *Antimicrob Agents Chemother* 43**,** 1062-1066.

Lozano, C., Aspiroz, C., Saenz, Y., Ruiz-Garcia, M., Royo-Garcia, G., Gomez-Sanz, E., Ruiz-Larrea, F., Zarazaga, M., and Torres, C. (2012). Genetic environment and location of the *lnu*(A) and *lnu*(B) genes in methicillin-resistant *Staphylococcus aureus* and other *Staphylococci* of animal and human origin. *J Antimicrob Chemother* 67**,** 2804-2808.

Malhotra-Kumar, S., Lammens, C., Piessens, J., and Goossens, H. (2005). Multiplex PCR for simultaneous detection of macrolide and tetracycline resistance determinants in *Streptococci*. *Antimicrob Agents Chemother* 49**,** 4798-4800.

Murakami, K., Minamide, W., Wada, K., Nakamura, E., Teraoka, H., and Watanabe, S. (1991). Identification of methicillin-resistant strains of *Staphylococci* by polymerase chain reaction. *J Clin Microbiol* 29**,** 2240-2244.

Ng, L.K., Martin, I., Alfa, M., and Mulvey, M. (2001). Multiplex PCR for the detection of tetracycline resistant genes. *Mol Cell Probes* 15**,** 209-215.

Sutcliffe, J., Grebe, T., Tait-Kamradt, A., and Wondrack, L. (1996). Detection of erythromycin-resistant determinants by PCR. *Antimicrob Agents Chemother* 40**,** 2562-2566.

Vakulenko, S.B., Donabedian, S.M., Voskresenskiy, A.M., Zervos, M.J., Lerner, S.A., and Chow, J.W. (2003). Multiplex PCR for detection of aminoglycoside resistance genes in *enterococci*. *Antimicrob Agents Chemother* 47**,** 1423-1426.

Wang, J., Lin, D.C., Guo, X.M., Wei, H.K., Liu, X.Q., Chen, X.J., Guo, J.Y., Zeng, Z.L., and Liu, J.H. (2015a). Distribution of the Multidrug Resistance Gene *cfr* in *Staphylococcus* Isolates from Pigs, Workers, and the Environment of a Hog Market and a Slaughterhouse in Guangzhou, China. *Foodborne Pathog Dis* 12**,** 598-605.

Wang, Y., Lv, Y., Cai, J., Schwarz, S., Cui, L., Hu, Z., Zhang, R., Li, J., Zhao, Q., He, T., Wang, D., Wang, Z., Shen, Y., Li, Y., Fessler, A.T., Wu, C., Yu, H., Deng, X., Xia, X., and Shen, J. (2015b). A novel gene, *optr*A, that confers transferable resistance to oxazolidinones and phenicols and its presence in *Enterococcus faecalis* and *Enterococcus faecium* of human and animal origin. *J Antimicrob Chemother* 70**,** 2182-2190.
